# Periodontal Health in Patients with Self-Ligating Brackets: A Systematic Review of Clinical Studies

**DOI:** 10.3390/jcm11092570

**Published:** 2022-05-04

**Authors:** Alexandru Mester, Florin Onisor, Anca Stefania Mesaros

**Affiliations:** 1 Department of Oral Health, University of Medicine and Pharmacy “Iuliu Hatieganu”, 400012 Cluj-Napoca, Romania; mester.alexandru@umfcluj.ro; 2 Department of Maxillofacial Surgery and Implantology, University of Medicine and Pharmacy “Iuliu Hatieganu”, 400012 Cluj-Napoca, Romania; 3 Department of Dental Propaedeutics and Aesthetics, University of Medicine and Pharmacy “Iuliu Hatieganu”, 400012 Cluj-Napoca, Romania; ancames@yahoo.com

**Keywords:** self-ligating bracket, orthodontic attachment, fixed appliance, periodontal health, periodontal disease

## Abstract

Background and objectives: The aim of this systematic review with meta-analysis is to assess the available evidence from human clinical studies of using self-ligating brackets compared to conventional brackets in maintaining periodontal health. Materials and methods: The protocol details were registered in the PROSPERO database (CRD42022302689). This review was performed under the PRISMA guidelines. The electronic search was performed in PubMed, Scopus, Web of Science and grey literature databases, as well as manual searches to find relevant articles published until January 2022. The inclusion criteria consisted of human clinical studies which reported the use of fixed orthodontic treatment with self-ligating brackets (SLBs) or conventional brackets (CBs) in maintaining periodontal health. Results: A total of 453 studies were imported into the Covidence Platform from the databases. Of these, six articles met the inclusion criteria. For plaque index, statistical significance was achieved for SLBs compared to CBs (0.31 (95% CI (0.15 to 0.48), *p* = 0.0001). For gingival index, probing depth and bleeding on probing no statistical significance was achieved. None of the included studies assessed clinical attachment level. Conclusions: The present systematic review with meta-analysis was considered to provide relevant data on periodontal health during orthodontic treatment in patients with SLBs in comparison with patients wearing CBs. Our findings indicated that SLBs are not superior to CBs in terms of periodontal health.

## 1. Introduction

A fixed orthodontic appliance is the most common method in correcting various types of malocclusions [1]. However, the presence of fixed orthodontic appliances on dental surfaces can influence oral hygiene; this is because of their design where fixed orthodontic appliances are retentive and facilitate the bacterial colonization [2]. The easier accumulation of biofilm, plaque and food residues increase the microbial contamination and, over time, may lead to the development of white spot lesions, caries, gingivitis and exacerbate periodontal disease [3,4]. This has led researchers to conclude that orthodontic therapy in combination with poor oral hygiene habits will lead to serious damage of the periodontium. Studies have previously shown that some clinical measures such as plaque index (PI), gingival index (GI), probing depth (PD) and bleeding on probing (BOP) increase their values following the placement of orthodontic appliances [5,6,7,8].

Orthodontic bracket systems have developed along with the progress in knowledge and technology of orthodontics, and their differences in design seem to influence the disruption of microbial environment [1]. Some designs, such as the conventional brackets, are considered to provide more retentive sites than others, thus increasing the number of microbial species and resulting in infection of the periodontal tissues, while others, such as self-ligating brackets, are considered to be less retentive and therefore more friendly towards the periodontium [9,10].

Self-ligating bracket (SLB) systems are being used more frequently, and they have been reported as having better force distribution toward the periodontal ligament tissues as compared to conventional preadjusted edgewise appliance (CB) systems [9,10,11,12]. This can be attributed to the fact that SLB systems present a low-friction force which does not decrease because of ligation material changes, such as the use of elastomeric materials in the oral cavity [9,10,11,12]. Current literature is inconclusive whether SLBs is superior to CBs. The meta-analysis of Yang et al. [11] aimed to compare weather SLBs can relieve discomfort and promote oral hygiene. The authors concluded that SLBs are not superior to CBs. Similar findings were reported by Arnold et al. [12] concerning the periodontal status in patients with different bracket systems designs.

Therefore, the aim of this systematic review is to present an update of the available evidence from clinical studies on the performance of self-ligating brackets compared to conventional brackets in maintaining periodontal health.

## 2. Materials and Methods

### 2.1. Protocol Registration

This systematic analysis was written according to the PRISMA guidelines [13]. A priori, the protocol details were submitted and registered in the PROSPERO database (code number CRD42022302689). The focused question of the present study was: “In patients following orthodontic treatment, what is the benefit of using self-ligating brackets compared to conventional brackets in maintaining periodontal health?”.

### 2.2. Eligibility Criteria

The inclusion criteria were established according to PICOS (Participants, Interventions, Comparison, Outcome, Study design) framework:

- Participants: Patients in good systemic health in treatment with fixed orthodontic brackets;
- Interventions: Fixed orthodontic treatment with self-ligating brackets (SLBs);
- Comparison: Fixed orthodontic treatment with conventional brackets (CBs) with steel/elastomeric ligature;
- Outcome: Changes in periodontal parameters recorded throughout orthodontic treatment phase (probing depth (PD), clinical attachment loss (CAL), bleeding on probing (BOP), Plaque Index (PI), Gingival Index (GI);
- Study design: clinical trial, randomized clinical trial (RCT), controlled clinical trial (CCT), prospective studies.

The following exclusion criteria were applied: patients with systemic diseases; in vitro studies, studies on experimental animal; reviews, case reports, letters to the editor; missing/uncompleted data regarding periodontal parameters; insufficient data that could not be used for the review; and articles published in other languages than English.

### 2.3. Search Process

The electronic literature search was conducted by two independent reviewers (A.M. and A.S.M.) on PubMed, Scopus, Web of Science. A grey literature search was conducted in the OpenGrey and ClinicalTrials.gov database. In addition, a manual search in journals specialized in orthodontics and periodontics has been carried out (American Journal Of Orthodontics And Dentofacial Orthopedics, European Journal Of Orthodontics, The Angle Orthodontist, The Korean Journal Of Orthodontics, Progress In Orthodontics, International Orthodontics, Journal Of Clinical Orthodontics, Journal Of Adhesive Dentistry, Orthodontics And Cranio-Facial Research, Journal Of Orofacial Orthopaedics, Dental Press Journal Of Orthodontics, Archives Of Orofacial Sciences, Seminars In Orthodontics, Journal Of The World Federation Of Orthodontists, Journal Of Orthodontic Science, Contemporary Clinical Dentistry, Turkish Journal Of Orthodontics, Australasian Orthodontic Journal, Journal Of Orofacial Sciences, Orthodontic Waves, Journal of Periodontal Research, Journal of Periodontology, Journal of Clinical Periodontology, Journal of Indian Society of Periodontology, International Journal of Periodontics and Restorative Dentistry, European Journal of Oral Sciences, Clinical Oral Investigations, Journal of Dental Research, Journal of Dentistry, Oral Medicine, Oral Pathology, Oral Radiology).

The electronic search was performed until January 2022 to identify relevant articles, using the following search strategy: (“self-ligating bracket” OR “bracket ligation” OR “conventional bracket” OR “elastomeric ligature” OR “wire ligature”) AND (“periodontal disease” OR “periodontitis” OR “periodontal health” OR “periodontal lesion” OR “periodontal bleeding” OR “periodontal inflammation” OR “gingivitis” OR “gingival health” OR “gingival lesion” OR “gingival bleeding” OR “gingival inflammation”). Firstly, titles and abstracts were scanned and selected for potentially relevant articles. Then, relevant articles were examined in full-text and those who fulfill the inclusion criteria were downloaded. If any disagreements were present, a third reviewer intervened with an investigation. The level of agreement between reviewers was established using Kappa coefficient. 

### 2.4. Data Extraction

The following data from the included studies were taken: first author, country of origin, design of the study, details concerning the number of participants, interventions, aims of the studies, periodontal indices that were followed, follow-up intervals, as well as the outcomes.

### 2.5. Risk of Bias Assessment 

Articles included in this systematic review were one RCT and 5 prospective studies. In regards to this, the quantification of risk of bias (RoB) was determined via ROBINS-I tool [14]. Each study was assessed in accordance with the following domains: confounding, participants, interventions, deviations from the intended interventions, missing data, and measurements of outcome, selection of the reported outcomes. The judgment of the RoB was evaluated as low (low risk for all fields), moderate (low/moderate for all fields), serious (serious risk in at least one field, but not critical in any field) or critical (critical risk in at least one field). Two independent reviewers (A.M. and F.O.) evaluated the RoB for the included studies, and if any disagreement was present, a third independent reviewer (A.S.M.) resolved the issue. 

### 2.6. Statistical Analysis

This analysis used means, standard deviations and percentages as outcome measurements. The heterogeneity of the studies was assessed using Cochrane Q test and the I^2^ index, at a significance level at 0.05. I^2^ was considered to be low for values ≤ 25%, moderate for values > 25 ≤ 50%, and high for values > 50% [15]. The 95% confidential interval (CI) and weighted means (WM) were calculated using random effect for continuous data between CBs + steel ligatures and SLBs. The results were depicted from forest plots. The program Review Manager (RevMan) (Computer program) (Version 5.4.1, The Cochrane Collaboration, 2020) was used for statistical analysis.

## 3. Results

### 3.1. Study Selection 

**Figure 1:**
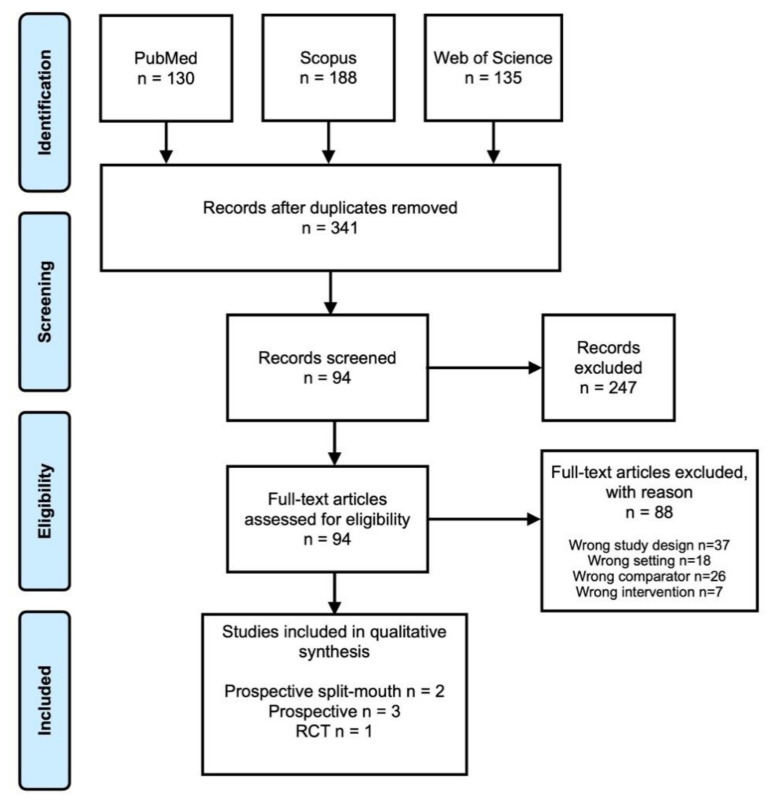
Prisma flowchart.

A total of 453 studies were imported into the Covidence Platform from the PubMed, Scopus, Web of Science databases. After removing 112 duplicates, a total of 341 articles were screened title and abstract, 247 studies were found to be irrelevant as they were not fitting with aims of our study, 94 studies were identified for full-text assessment, and only 6 articles [10,16,17,18,19,20] met all required inclusion characteristics at the end of the full text analysis. A brief description of the selection process, as well as the reasons for the exclusion of articles during the full-text assessment is shown in a flow diagram, according to the PRISMA statement (Figure 1). 

### 3.2. Study Characteristics

#### 3.2.1. Description of Included Studies 

From the selected studies, five were on teenage populations [10,16,17,18,19], while one [20] was on an adult population with the added characteristic of chronic periodontitis pathology associated. All studies included the measurement of periodontal indices in order to assess changes in the periodontal status due to the fixed orthodontic appliances. Other aims were the assessment of dental plaque retention, status of oral hygiene and halitosis.

#### 3.2.2. Study Design

The studies included were published between 2013 and 2021 and were conducted in Turkey, China, Brazil, and a mixed team from USA and Australia (Table 1). In regards to the study design, there were two prospective split-mouth studies [16,18], three prospective studies [10,19,20], and one randomized clinical trial [17]. The measurements of periodontal indices were performed before the bonding of brackets and at different follow-up intervals afterwards: 3 studies performed measurements 1 week after bonding [10,16,19], 3 studies performed measurements 4–5 weeks [10,18,19] or 30 days [18] after bonding, and 3 studies performed measurements 60 days after bonding [18,19,20]. However, the periodontal indices studied were not homogenous. Among the selected studies, we found that four studies used Plaque Index [16,18,19,20], three studies used for Gingival Index [18,19,20], and two studies used Probing Depth and Bleeding on Probing [19,20]. The RCT from Chhibber and coworkers [17] presented the following particularities: periodontal parameters were assessed only on the maxillary second premolar in comparison with the other studies that assessed all teeth available; another particularity was the comparison with patients using the treatment with Clear Aligners. 

#### 3.2.3. Characteristics of Participants 

In total, 323 patients (115 men and 82 women; of which two studies with 126 participants failed to describe the gender) with ages between 12 and 40 were included. The sample sizes varied from 16 to 110 patients. The study from Baka [16] was conducted exclusively on male patients with a mean age of 14.2 years, and the study of Wang [20] only addressed adult patients with ages between 18 and 40. In addition, the adult population from Wang’s study presented chronic periodontitis, while patients from the other five studies were addressing teenagers with no presence periodontal pathology. Individual characteristics of the included studies are presented in Table 1.

### 3.3. Risk of Bias Assessment

**Figure 2:**
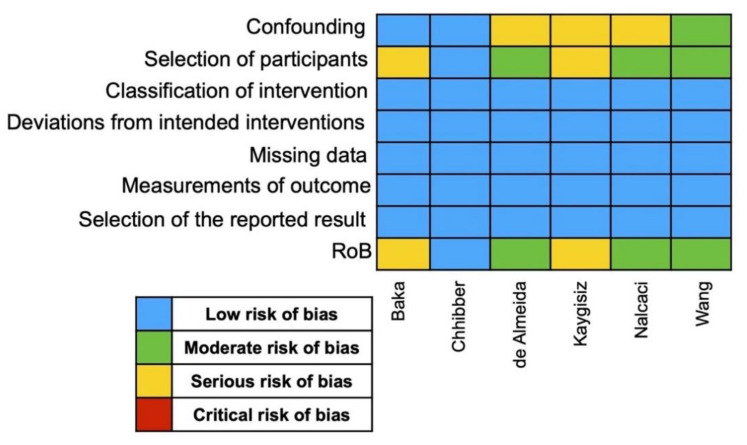
RoB assessment.

The RoB results is presented in Figure 2. Overall, the RCT from Chhibber and coworkers [17] was the only one with low RoB. On the other hand, three studies had moderate RoB [10,18,20], and two studies presented serious RoB [16,19]. The RoB analysis is shown in Figure 2. 

### 3.4. Periodontal Indexes Assessment

**Figure 3:**
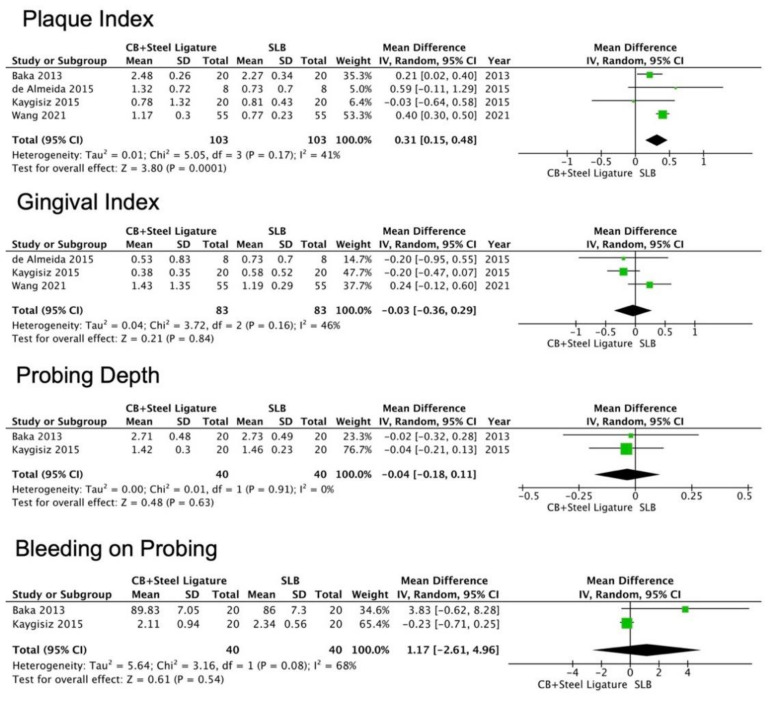
Forest plots for plaque index, gingival index, probing depth and bleeding on probing. Plaque index, gingival index and bleeding on probing were reported in percentages (%). Probing depth was reported in millimeters (mm).

For PI index, from the four studies included, a higher trend towards SLBs with statistical significance compared to CBs was observed, having WM of 0.31 (95% CI (0.15 to 0.48), *p* = 0.0001) (Figure 3). For GI index, from three studies included, no statistical significance was achieved. In addition, for the PD and BOP index, from two studies included, no statistical significance was found. For the CAL index, none of the included studies assessed this parameter.

## 4. Discussion

The present systematic review with meta-analysis was considered to provide relevant data on periodontal health during orthodontic treatment in patients with SLBs in comparison with patients wearing CBs. After an exhaustive and comprehensive literature search and evaluation, six studies were recruited in this systematic review, among which four articles were statistically included for a pertinent quantitative analysis. 

According to the consensus published by Chapple et al. in 2017 [21], periodontal health represents the absence of any clinical inflammation to the periodontium. Therefore, the authors have stated that periodontal health must be assessed at the patient and at the site level. From the reported differences in the treatment modalities, it is difficult to make accurate comparisons between the included studies. Therefore, it must be kept in mind that the aim of our systematic review was to see if SLBs may offer a better result in terms of periodontal health compared to CBs. 

Chapple et al. [21] defined clinical gingival health on an intact periodontium as the absence of BOP, erythema, edema, with no CAL and a PD between 1 and 3 mm. Other features of clinical gingival health are on a reduced periodontium (which has as distinct features attachment and bone loss) and following successful treatment of periodontitis (presence of reduced clinical attachment and bone level). From the included studies, none them have mentioned a clear case definition about periodontal health.

If we are to compare our systematic review with other similar papers, the differences are quite disparaging. As mentioned by Yang and coworkers [11], the design of SLBs is able to reduce microbial colonization and promote oral health due to the configuration without ligature. Choices of bracket types in the clinical settings are still a debate in the approach of orthodontic patients. Fleming and collaborators [22] concluded that SLBs and CBs are similar in terms of treatment efficiency. Nevertheless, with SLBs being free of ligation by either elastomeric or steel ligatures, the archwire has more play in slots of SLBs than in CBs, which translates in a lower frictional force and thus might exert lighter forces to teeth in the levelling and alignment phase [23,24]. In addition, in everyday practice, self-ligating brackets are believed to provide the benefits of reducing chair time, an advantage for both practitioner and patient; to shorten treatment duration; to deliver less pain-related to tooth movement; and to favor a better oral hygiene, as they are considered to have a poorer biostability [21].

The meta-analysis of Arnold and coworkers [12] found that the short-term effects (4–6 weeks after brackets placement showed no superiority to none of the brackets. In the interval time of 3–6 months of orthodontic treatment, evidence was very low in favor of SLBs in terms of PI (WMD 0.14 (95% CI 0.0, 0.28); *p* = 0.05). For GI and PD parameters, no statistical significance was achieved for both CBs and SLBs. Authors have mentioned that disparity across the included studies did not allow for clear conclusions to support the advantages of over CBs in periodontal health in adolescents’ patients. It is worth mentioning that Arnold et al. [12] included RCTs; as far as we have seen, we had a common ground of four articles [10,16,18,19], of which, from our perspective, the articles were not registered as RCT in the trial’s portal. 

The meta-analysis written by Maizeray and coworkers [25] assessed the efficiency of CBs, passive and active SLBs. The authors concluded that no differences between the three types of brackets had been seen. In terms of periodontal indices, they found less BoP for passive SLBs compared to CBs after 4–5 weeks after bonding (WMD −0.10 (95% CI −0.12 to −0.08); *p* = 0.00001); the results were taken from 2 articles [10,19].

Another interesting meta-analysis was published by Huang and coworkers [26] who assessed the effects of fixed orthodontic brackets (CB and SLB) on oral malodor. In the PI parameter, CBs showed no statistical significance compared with the control group at the initial visit and 1 week after bonding. At 1 month after bonding, statistical significance was achieved for PI parameter (WMD 0.24 (95% CI 0.05–0.43); *p* = 0.01) and for GI parameter (WMD 0.30 (95% CI 0.06–0.54); *p* = 0.01). In regards to the PD parameter, no statistical significance had been achieved over the 3-month period after bonding. In the end, the authors concluded that the available evidence was weak, and fixed orthodontic treatment represented a risk factor for malodor at 1 week after bonding, which was independent of PI changes. In addition, SLBs were able to better control the malodor compared to CBs. 

The meta-analysis of Yang and coworkers [11] aimed to compare SLBs and CBs and whether they can promote oral health and relieve discomfort. The authors found out that SLBs are not superior to CBs in promoting oral health and supreme discomfort. The statistical analysis for the PI parameter showed that passive SLBs and CBs did not differ significantly (WMD 0.04, 95% CI (0.30,0.22), *p* = 0.07). Verrusio et al. [27] published a systematic review on the effects of orthodontic treatment of periodontal inflammation. They mentioned that fixed orthodontic therapy may increase periodontal inflammation during and after treatment. In addition, the authors indicate that accumulation and composition of subgingival microbiota may increase periodontal inflammation, which results in higher BoP scores (no statistical analysis was available).

The present review has several limitations. Although the literature search was extensive, only six articles corresponded to the inclusion criteria, and four of the studies were included in the meta-analysis, resulting in a deficient statistical analysis. The results of our research suggest that there is no statistically significant difference to be found between SLBs and CBs in terms of periodontal health. The lack of heterogeneity among clinical studies makes the reported results to be interpreted with caution. Therefore, in the future, more methodologically sound clinical trials should be reported with precise and common guidelines, as to improve the quality of research, allowing for more comprehensive meta-analyses and to make certain aspects of research including method of randomization and allocation concealment more transparent.

## 5. Conclusions

The findings of our systematic review and meta-analysis indicated that self-ligating brackets are not superior to conventional brackets in terms of periodontal health. In future, well-designed, prospective multicentric studies should be conducted, with respect to the periodontal indexes and follow-up timelines.

## Figures and Tables

**Table 1:** Characteristics of the included studies.

Author. Country. Reference	Study Design	Participants	Interventions	Aims	Periodontal Indexes	Follow-Up	Outcomes
Baka, Turkey [16]	Prospective, split-mouth	*n* = 20 Male: 20 Mean age: 14.2 ± 1.5 years	SLBs (Damon Q; Ormco, Orange, Calif) CBs (Roth-equilibrium 2, 722-341; Dentaurum, Pforzheim, Germany) ligated with 0.010-in conventional stainless steel ligature wires	Dental plaque retention Microbial flora Clinical periodontal parameters	PI PD BOP	Before bonding 1 week after bonding 3 months after bonding	Similar significant increase of periodontal measurements in both groups during first 3 months of treatment. Differences were not statistically significant.
Chhibber, USA and Australia [17]	RCT	*n* = 71 Male: 41 Female: 30 Mean age: 15.6 years	SLBs (Carriere, Carlsbad, Calif (*n* = 22) CBs (Ortho Organizers Inc., Carlsbad, CA) with elastomeric ligated brackets (*n* = 22) CA (Align Technology, San Jose, Calif) (*n* = 27)	Oral hygiene assessment by periodontal measurements	PI GI PBI	Before treatment after 9 months of treatment After 18 months or completion of treatment	In the short term the CA group participants had better GI and PBI scores than the fixed appliance groups. No evidence of any significant difference in the oral hygiene levels among CAs, SLBs, and CBs after 18 months of treatment.
de Almeida Cardoso, Brazil [18]	Prospective, Split-mouth	*n* = 16 Age: 12–16 years	SLBs-Portia model (3M, São José Rio Preto, São Paulo, Brazil), with a NITI slot locking mechanism CBs -Kirium model (Abzil-3M, São José Rio Preto, São Paulo, Brazil), with metallic ligatures	Periodontal response	VPI GBI CAL	Before treatment After 30 days of treatment After 60 days of treatment After 180 days of treatment	No significant differences for either one of the variables.
Kaygisiz, Turkey [19]	Prospective	*n* = 60 Male: 32 Female: 28 Age: 12–18 years	SLBs; (F1000, Leone SpA, Sesto Fiorentino, Florence, Italy) (*n* = 20) CBs (Avex MX, Opal Orthodontics, South Jordan, Utah with steel ligatures) (*n* = 20) Control group (*n* = 20)	Halitosis Periodontal status	PI GI BOP PD	1 week before bonding immediately before the placement of brackets 1 week after bonding 4 weeks after bonding 8 weeks after bonding	The SLBs do not have an advantage over CBs with respect to periodontal status and halitosis.
Nalcaci, Turkey [10]	Prospective	*n* = 46 Male: 22 Female: 24 Age: 11–16 years)	SLBs (Damon Q; Ormco, Glendora, Calif) CBs (Mini Taurus; Rocky Mountain Orthodontics, Denver, Colorado with elastomeric ligatures)	Halitosis, Periodontal status Microbial colonization.	GI PI BOP	Before bonding 1 week after bonding 5 weeks after bonding	GI and PI values of the SLBs group were lower. The BOP values of both groups showed significant differences at all the time intervals, but in the SLBs group, there were no significant differences between the 1 week and 5 weeks periods. Periodontal parameters and halitosis results were higher in the CBs group than in the SLBs group.
Wang, China [20]	Prospective	*n* = 110 Age: 18–40 years chronic periodontitis	CBs-control group SLBs-research group	Periodontal tissues Inflammatory factors	CAL SBI GR PLI TM	Before treatment 2 months after treatment	The CAL, SBI, PLI, TM and GR of both groups delivered much better results 2 months after treatment. SLBs indicated greater changes than the CBs group.

BOP: bleeding on probing; CA: Clear Aligners; CAL: clinical attachment level; CB: conventional bracket; GBI: gingival bleeding index; GI: gingival index; GR: gingival recession; PBI: papillary bleeding index; PD: probing depth; PI: plaque index; SLB: self-ligating bracket; TM: tooth mobility; VPI: Visible plaque index.

## Data Availability

Not applicable.
